# Effects of isomaltodextrin in postprandial lipid kinetics: Rat study and human randomized crossover study

**DOI:** 10.1371/journal.pone.0196802

**Published:** 2018-05-01

**Authors:** Ryodai Takagaki, Yuki Ishida, Tsuyoshi Sadakiyo, Yoshifumi Taniguchi, Takeo Sakurai, Hitoshi Mitsuzumi, Hikaru Watanabe, Shigeharu Fukuda, Shimpei Ushio

**Affiliations:** R&D Center, Hayashibara Co., Ltd., Okayama, Japan; The University of Tokyo, JAPAN

## Abstract

Isomaltodextrin (IMD) is a novel dietary fiber-like polysaccharide: a type of α-glucan produced from starch using enzymes derived from microorganisms. The results of cohort studies show that dietary fiber can prevent cardiovascular disorders caused by lifestyle-related diseases such as metabolic syndrome. Inhibition of excess fat absorption by dietary fiber is known to be one of the mechanisms, and it is also known that the actions of dietary fiber vary depending on factors such as its structure or origin. Thus, we investigated the inhibitory actions of IMD on fat absorption, and analyzed its mechanism of action. In rats, the absorption of fat given by gavage was significantly lower at 1, 2, and 6 hours after IMD administration than after vehicle administration. In humans, IMD was associated with a lesser increase in blood triglycerides in subjects whose blood triglycerides were otherwise apt to rise. We also found by in vitro emulsion studies that IMD, which had no effect on digestive enzyme activity or emulsion formation, stabilized the micro size micelle by inducing enlarged micelle particle size and increased zeta potential. In conclusion, the mechanism of inhibition of fat absorption by IMD may be a delay in micelle particles accessing the intestinal epithelium through changes in the surface structure and the physical properties of the micelle particles.

## Introduction

Obesity has been increasing worldwide at an accelerated rate since the year 2000, and the prevalence rate of obesity in the aged has become an issue that cannot be ignored [1, 2]. Excess fat intake is the main cause in developed countries, requiring prompt improvement of nutrient balance.

Continuous lifestyle-related clinical conditions, such as metabolic syndrome, caused by excess fat intake may cause life-threatening lesions [3, 4] that involve serious damage to blood vessels. Excess fat intake has been observed to be a risk factor for vascular occlusion accompanied by infarction disorders, due to increased adipose tissue mass [4, 5].

While health damage caused by an excessive intake of fat has been increasing, it is known that dietary fiber inhibits absorption of excessive nutrients [6]. Intake of dietary fiber is recommended by the report “Diet, Nutrition and the Prevention of Chronic Diseases” published by the World Health Organization and the Food and Agriculture Organization as a means to prevent current lifestyle-related diseases [7, 8]. Among its actions, dietary fiber has been reported to inhibit fat absorption and significantly prevent development of vascular diseases [9, 10].

Extracted/processed dietary fibers are now commercially available as dietary fiber sources besides plant-based foods, and they are classified as insoluble or water soluble [11]. Most of these fibers are directly extracted from plants or made from starch.

**Fig 1:**
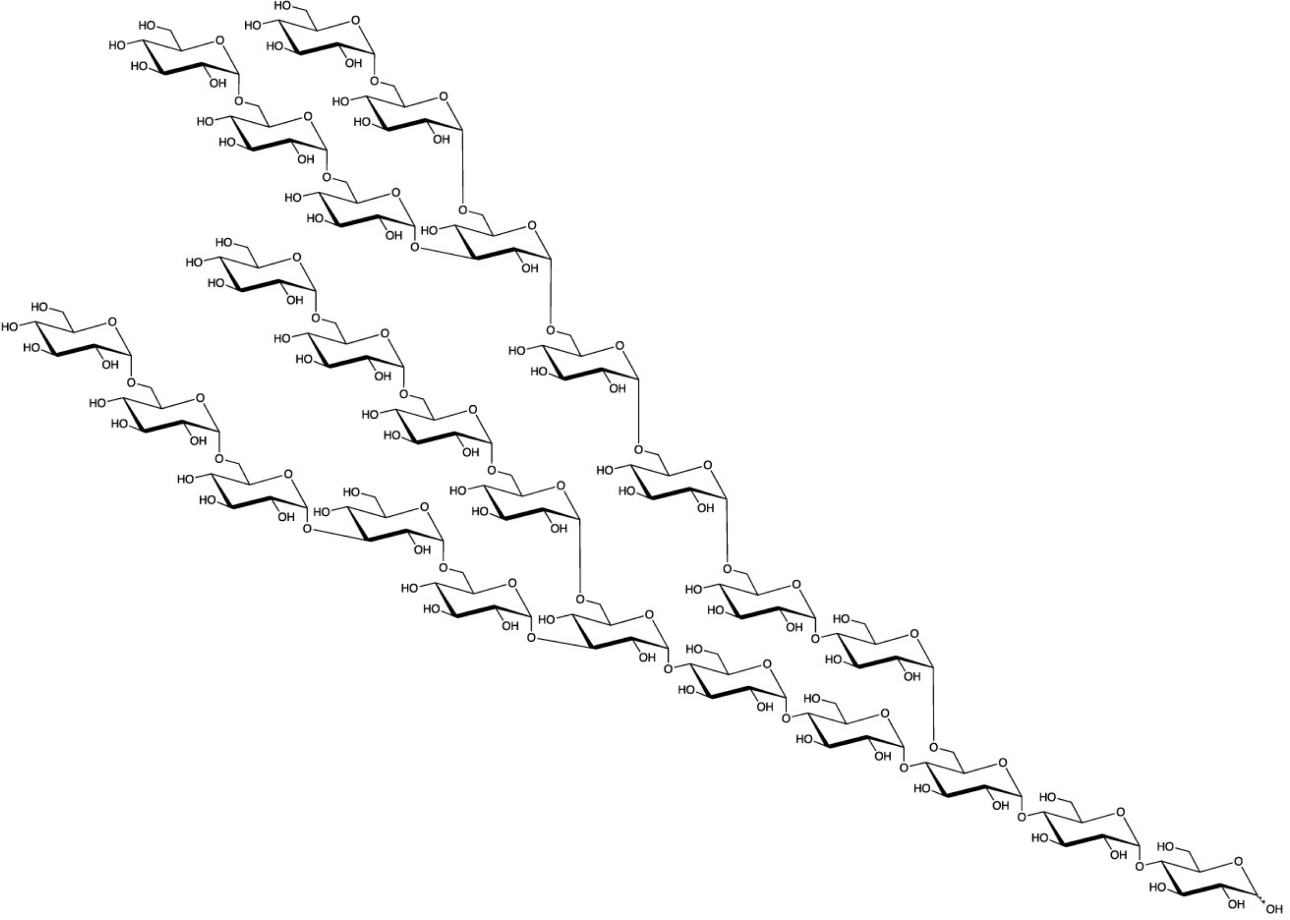
The putative structure of IMD.

Isomaltodextrin (IMD) is a water-soluble dietary fiber produced enzymatically from starch using α-glucosyltransferase and α-amylase derived from *Paenibacillus alginolyticus* PP710 [12]. IMD includes approximately 17% α-1 glucosidic linkages, 3% α-1,3-glucosidic linkages, 19% α-1,4-glucosidic linkages, 49% α-1,6-glucosidic linkages, 7% α-1,3,6-glucosidic linkages, and 5% α-1,4,6-glucosidic linkages. The weight-average molecular weight is 5,000 (Fig 1). Resistant maltodextrin (RMD) and polydextrose are also derived from starch, but IMD has a unique chemical structure, consisting of only α-linkages and including many α-1,6-linkages.

IMD intake has been associated with effects similar to those of other types of dietary fiber, such as suppression of blood glucose elevation [13] and improvement of bowel movements [14, 15].

Since dietary fiber is known to prevent absorption of excess fat, IMD is also expected to have such an effect. Therefore, we examined the preventive effect of IMD on fat absorption by using a fat-loading test in rats and humans. We also investigated the mechanism of its suppressive effect on fat absorption in vitro.

## Materials and methods

### Chemicals

Olive oil, bovine gall powder, triolein, lecithin (egg yolk), sodium taurocholate, catechin mixture, and oleic acid were purchased from Wako Pure Chemical Industries, Ltd. (Osaka, Japan). Lipase, glyceride monooleate, and cholesterol were purchased from Sigma-Aldrich Corporation (Tokyo, Japan). Epigallocatechin gallate hydrate (EGCG) was purchased from Tokyo Chemical Industry Co., Ltd. (Tokyo, Japan). Oleic acid-glyceryl monooleate mixture was purchased from MP Biomedicals (Tokyo, Japan). Indigestible dextrin was purchased from Matsutani Chemical Industry Co., Ltd. (Hyogo, Japan). Pectin was purchased from Sansho Co., Ltd. (Osaka, Japan). IMD was produced by Hayashibara Co., Ltd. (Okayama, Japan)[12].

### Animal experiments

7-wk-old male Wistar rats (Charles River Laboratories, Kanagawa, Japan) were housed individually in plastic cages. The animal room was controlled at a temperature of 23 ± 1°C and 40%–70% humidity in a 12-hour light/12-hour dark cycle (lights on from 7:00 am to 7:00 pm). Rats were fed a commercial solid diet (CE-2: CLEA Japan Inc., Tokyo, Japan) and water ad libitum. Healthy animals were used in the experiments after a 2-week acclimation period.

Animals were divided into 3 groups of 6 animals each (control group, low IMD group, and high IMD group) with a similar mean body weight, and used for a fat-loading test after an overnight fast. The control group was administered a mixture of 17 mL of a commercially available lipid emulsion made from soybean oil (INTRALIPOS: Intralipos Injection 10%, Otsuka Pharmaceutical Factory, Inc., Tokushima, Japan) and 3 mL of water by gavage under unanesthetized conditions. The two IMD groups were administered a mixture of 17 mL of INTRALIPOS and 3 mL of a 7.6% IMD solution (low IMD group) or a 38% IMD solution (high IMD group). The dose of control lipid was 1.5 g/kg-body weight (bw) and the dosing volume was 17.7 mL/kg-bw. The dose of IMD was 1.0 g/kg-bw for the high IMD group and 0.2 g/kg-bw for the low IMD group.

Blood was collected from the tail veins of the rats under unanesthetized conditions before and 1, 2, 3, 4, and 6 hours after administration of the lipid emulsions. Triglyceride (TG) levels in plasma were determined with Triglyceride E-test Kit (Wako Pure Chemical, Osaka, Japan).

This animal experiment was approved by the Animal Care and Use Committee of the R&D Center of Hayashibara Co., Ltd. (approval number: hb1602-04), and performed in accordance with the Regulations on Animal Experimentation at the R&D Center of Hayashibara Co., Ltd.

### Suppression of postprandial increase in human blood triglyceride

This human study was implemented to meet the requirements of CONSORT 2010 (S2 File).

#### Subjects

A total of 40 healthy Japanese volunteers (15 men and 25 women) aged from 20 to <70 years and with a fasting blood TG level from 30 to <150 mg/dL were recruited and registered as study subjects (by February, 2017) by the Medical Corporation Hokubukai Utsukushigaoka Hospital (Hokkaido, Japan). They were selected according to the criteria of S1 File. In this study, subgroup analysis was planned in advance in addition to all subjects analysis. Previously, similar studies were conducted in 13 subjects per group [16]. We assumed that subgroup analysis could be divided into two groups in this case study. We expected that the number of subjects in subgroup analysis was more than that of previous study. Therefore, we assigned 20 subjects per group to subgroup analysis considering dropouts, resulting in a total number of 40 subjects in the whole study. No formal power calculation was conducted in this study. All subjects received an adequate explanation of the purpose and procedures of the study and gave written informed consent. This study was performed in conformity with the Ethical Guidelines for Medical and Health Research Involving Human Subjects as well as the Declaration of Helsinki, was approved by the Ethical Committee at the Medical Corporation Hokubu-kai Utsukushigaoka Hospital (Study No. 16S131, January 19, 2017), and was registered in the clinical study database University Hospital Medical Information Network; ID: UMIN000026170. All human intervention studies were performed with consideration of the subjects' human rights and under the supervision of physicians in the indicated hospital.

#### Test diet

A corn soup consisting of 20.0 g of butter (Yotsuba Milk Products Co., Ltd., Hokkaido, Japan) and 13.9 g of lard (Megmilk Snow Brand Co., Ltd., Tokyo, Japan) and 200.0 g of corn potage soup (Nagoya Seiraku Co., Ltd., Aichi, Japan) was used as a high fat-loading diet containing 40 g of fat in total. Two test diets (the IMD diet and the placebo diet) were prepared before use. The IMD diet was a mixture of the above mentioned high fat-loading diet and 2.5 g of IMD (equivalent to 2.13 g of dietary fiber), while the placebo diet was the high fat-loading diet that did not contain IMD. We confirmed that the IMD diet and the placebo diet were indistinguishable in appearance, taste, and texture.

#### Study schedule and intake methods

This study was conducted using a double-blind, randomized, placebo-controlled crossover design. An oral fat-loading test was performed to screen the subjects who meet the eligibility criteria using the placebo diet. We selected the healthy subjects taking into account of the TG levels after fat-loading and other laboratory findings. Next, an assignment person who was not directly involved in the study randomly assigned the subjects to 2 groups (groups A and B) using random numbers, so that the groups had the same sex ratio and change in TG levels (ΔTG). The order of intake of the test diets was also assigned in each group (A and B). The Assignment Person sealed the assignment table for subjects and intake order and kept it until the fixation of all data.

The subjects of each group received the 2 test diets with intervals of not less 1 week between the diets. The subjects were instructed to spend time as usual without excess exercise and food intake or lack of sleep during the study period. The subjects fasted except for a small amount of water for more than 12 hours from the supper time (unified meal) on the night before the study until the next morning. On the morning of the study, blood was collected in the fasting state, and then the subjects consumed the IMD diet or placebo diet over 5 minutes. Blood was collected 2, 3, 4, and 6 hours after the intake. The subjects waited in a sitting position with no food and no drink from 1 hour before the intake of the test diet to the last blood collection.

#### Endpoints

Baseline TG and ΔTG were determined before and after intake as the primary endpoint. TG and ΔTG were compared between the test diets at the time point when the maximum TG concentration was reached in subjects receiving the placebo diet. As for changes over time of TG and ΔTG, the area under the curve (AUC and ΔAUC) as well as the maximum concentrations of each subject (C_max_ and ΔC_max_) were calculated and compared between the test diets. C_max_ and AUC were calculated using TG, ΔC_max_ and ΔAUC were calculated using ΔTG. AUC and ΔAUC were calculated based on trapezoidal rule.

Furthermore, subgroup analysis was performed in subjects with a relatively high risk of abnormal lipid metabolism for whom blood TG control should be considered, based on the C_max_ of TG in each subject receiving the placebo diet, as described in the protocol. Specifically, we selected subjects with a C_max_ of 200 mg/dL or more in a non-fasted condition for subgroup analysis.

Blood remnant-like particle cholesterol (RLP-cholesterol) was evaluated as a secondary endpoint in the same manner as TG.

The safety of the test diet was evaluated by a physician, based on conventional examinations of symptoms and adverse events.

### Inhibition of emulsification

The effect of IMD on emulsification was evaluated according to the methods reported by Koseki et al.[16] An emulsion was prepared by mixing 1 mL of olive oil and 11.5 mL of distilled water with 0.2% bovine gall powder (Wako Pure Chemical, Osaka, Japan), followed by sonication for 1 minute. This emulsion was mixed with an equal volume of 0.2 M Tris-HCl buffer (pH 8.0) as the negative control, 0.2 M Tris-HCl buffer (pH 8.0) containing 1% pectin as the positive control, or 0.2 M Tris-HCl buffer (pH 8.0) containing 10% IMD as the test substance; each were shaken at 37°C for 60 minutes at 80 strokes/minute. Samples were taken at 0, 15, 30, 45, and 60 minutes. After 100-fold dilution with 0.1% sodium dodecyl sulfate, the absorbance was measured at 500 nm.

### Inhibition of lipase activity

The effect of IMD on lipase activity was evaluated according to the methods reported by Narita et al [17]. An emulsion was prepared by mixing 80 mg of triolein, 10 mg of lecithin, 5 mg of sodium taurocholate, and 9 mL of 0.1 M HEPES buffer (pH 7.0), followed by sonication (250 sonifier: Blanson Ultrasonics, Danbury, CT, USA) for 10 minutes. Forty-microliter aliquots of this emulsion were mixed with 40 μL each of 1%, 2%, 5%, 10%, and 20% IMD aqueous solution, and incubated at 37°C for 30 minutes. Lipase was added to each mixture at a concentration of 0.4 mg/mL, and the resulting mixtures were incubated at 37°C for 30 minutes. The enzyme reaction was terminated by boiling for 30 minutes. Free fatty acids in the mixture were determined by using NEFA C-Test Wako (Wako Pure Chemical, Osaka, Japan). Epigallocatechin gallate (2 mM) was used as a positive control with reference to the report of Ikeda et al. [18], and 0.1 M HEPES buffer served as a negative control.

### Suppression of micelle breakdown

The effect of IMD on fatty acid micelles (after reaction with lipase) was evaluated according to the methods reported by Kishimoto et al. [19]. A micelle emulsion was prepared by mixing 0.5 g of oleic acid-glyceryl monooleate mixture and 0.125 g of sodium taurocholate with 5 mL of water or of 20% IMD solution or of 20% resistant maltodextrin. Each mixture was then sonicated (250 sonifier: Blanson Ultrasonics, Danbury, CT, USA) for 1 minute and then allowed to stand at 37°C. Samples were taken at 0, 15, 30, 45, and 60 minutes. After 400-fold dilution with 0.1% sodium dodecyl sulfate solution, the absorbance was measured at 500 nm. The particle size was measured with a Mastersizer 3000 laser diffraction sizer (Malvern Instruments Ltd, Worcestershire, UK).

### Evaluation of physical properties of micelles

Micelle suspensions were prepared with reference to the methods of Ikeda et al. [20]. Glyceryl monooleate (5 mM), oleic acid (10 mM), triolein (10mM), lecithin (5 mM), cholesterol (10 mM), and sodium taurocholate (10 mM) were suspended in 150 mM phosphate buffer (pH 7.0), followed by sonication for 5 minutes. After stabilization at 37°C for 24 hours, an aliquot of the emulsion was centrifuged with an ultracentrifuge (SCP70H2, Hitachi Koki Co., Ltd., Tokyo, Japan) at 100,000 g and 37°C for 1 hour to remove coarse fractions. This solution was used as the micelle solution.

Just before measurement, 0%, 10%, and 20% (g/mL) IMD solutions were prepared in 150 mM phosphate buffer (pH 7.0), and the micelle solution was 11-fold diluted with each of the IMD solutions. The zeta potential and micelle particle size were measured with a Zeta Sizer Nano ZS (Malvern Instruments Ltd., Worcestershire, UK). When measuring the zeta potential, the dielectric constant of the dispersion medium was set to 78.5 and the refractive index was set to 1.330. The viscosity was measured with a rotating viscometer (BrookField, Ontario, Canada) at 37°C.

### Statistical analysis

For the rat and human studies, all values are expressed as mean ± standard deviation (SD).

For the in vitro studies, the values in the figures and tables are shown as mean ± standard error (SE).

In the animal and in vitro studies, the data were analyzed by analysis of variance with a Dunnett's or Tukey-Kramer test to compare multiple groups. Differences were considered significant if the p value was <0.05. BellCurve for Excel software (Social Survey Research Information Co., Ltd., Tokyo, Japan) was used for the statistical analysis.

In the human study, the effects of timing and order of the intake of the test diets on the ΔAUC of TG were evaluated using analysis of variance to examine the adequacy of the crossover study. Statistical analysis was performed with the Wilcoxon signed-rank test, with a p value <0.05 (two-sided) being considered statistically significant. IBM SPSS Statistics 24 (IBM Corporation, Armonk, NY, USA) was used for statistical analysis.

## Results

### Fat-loading test in rat

**Fig 2:**
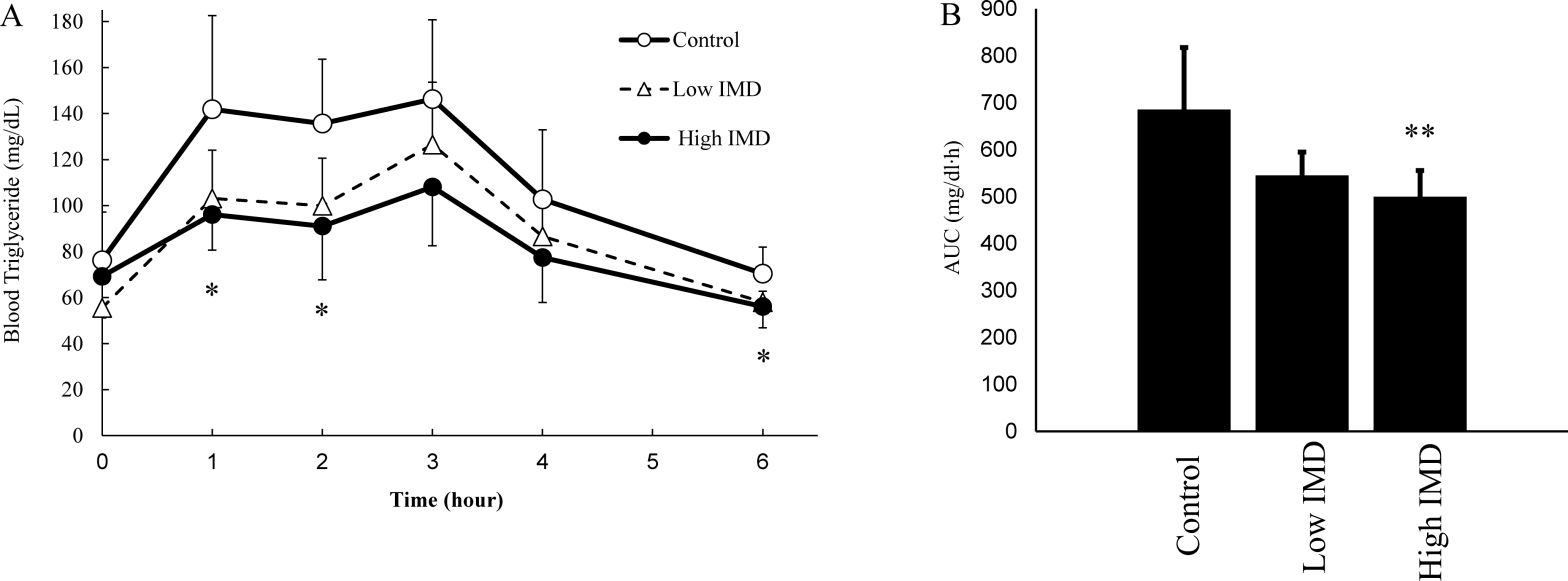
Triglyceride kinetics in blood. (A) Transition blood TG levels in time after lipid loading. (B) Area under the blood concentration-time curve. ○: Control (1.5 g/kg-bw oil); n = 6, △: Low IMD (1.5 g/kg-bw oil + 0.2 g/kg-bw IMD); n = 5, ●: High IMD (1.5 g/kg-bw oil + 1.0 g/kg-bw IMD); n = 6. Values are mean ± standard deviation (n = 5 or 6). Statistical analyses were performed using a Tukey-Kramer multiple comparison test (vs control: *p <0.05, **p <0.01).

Fig 2 shows the TG kinetics in blood after fat-loading in each group. A multiple comparison test was performed to examine the change in blood TG over time between groups, showing that there were significant differences at 1, 2, and 6 hours in the high IMD group compared with the control group. Though the low IMD group showed no significant differences at any of the time points compared with the control and high IMD groups, TG levels were lower in the low IMD group than in the control group. The AUC of TG was significantly lower in the high IMD group than in the control group. There were no significant differences in the AUC of TG between the low IMD group and the control group or the high IMD group.

### Suppression of postprandial increase in human blood triglyceride

#### Subjects

**Fig 3:**
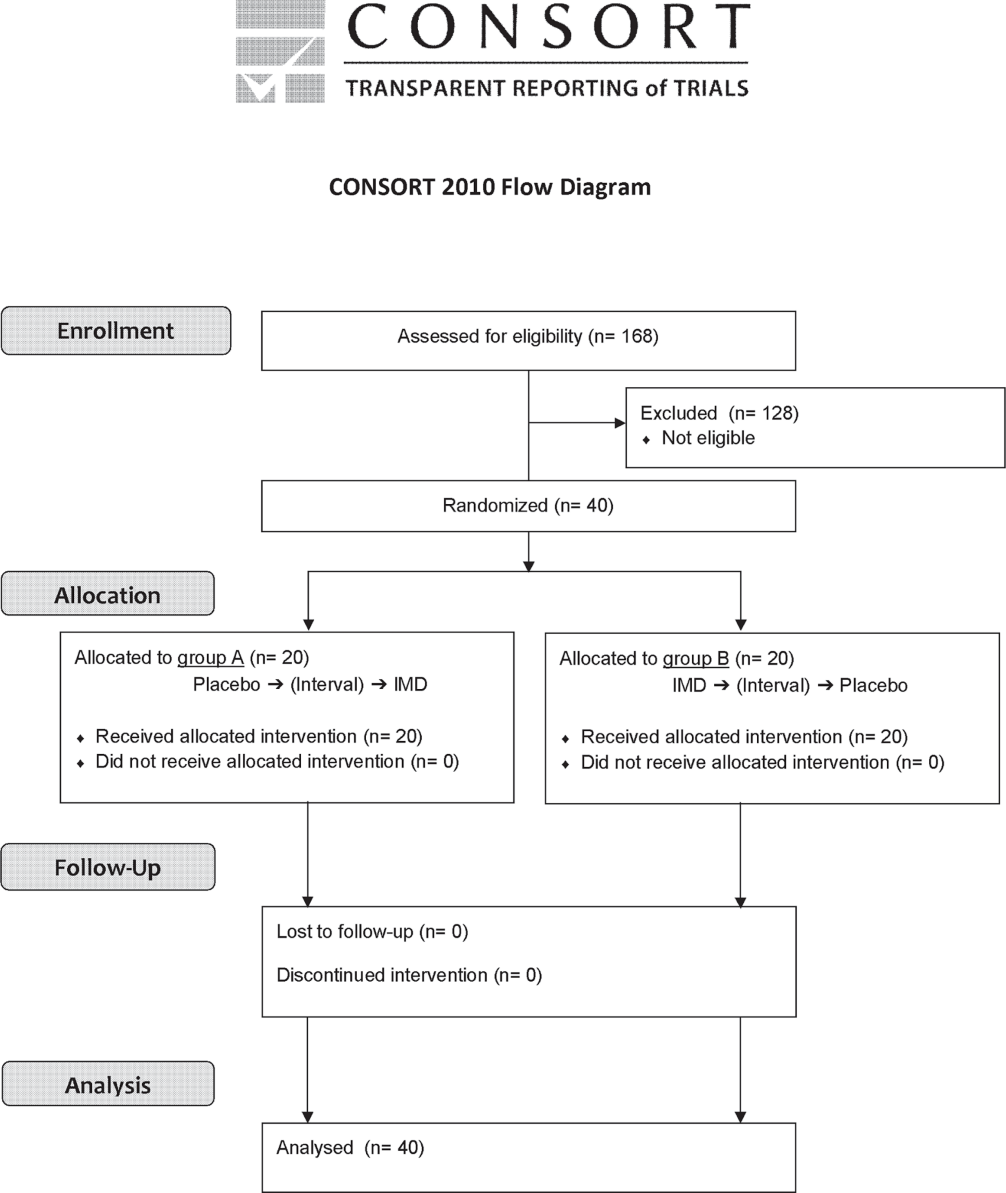
Trial flow chart.

Forty subjects who were screened from 168 candidates giving their informed consent were randomized to 2 groups of 20 subjects each (Fig 3). All subjects completed the study schedule (the study dates: February 27 and March 6, 2017), and no subjects withdrew or dropped out throughout the study period. In addition to the analysis of all subjects, we performed a subgroup analysis of 14 subjects who had a TG C_max_ of ≥ 200 mg/dL after consuming the placebo diet. The demographic characteristics of the subjects are shown in Table 1.

**Table 1:** Demographic characteristics of subjects.

	All subjects			Subgroup analysis subjects ^a)^		
	Total	A group	B group	Total	A group	B group
Male (n)	15	8	7	5	3	2
Female (n)	25	12	13	9	3	6
Total subjects (n)	40	20	20	14	6	8
Age (years old)	35.4±12.8	35.6±13.8	35.2±12.0	35.8±11.7	38.7±13.6	33.6±10.5
Height (cm)	163.4±7.6	164.6±8.1	162.2±7.1	162.4±7.3	164.6±8.5	160.7±6.3
Body weight (kg)	58.1±10.1	57.3±8.9	59.0±11.3	60.6±8.5	61.4±8.5	60.0±9.0
Systolic blood pressure (mmHg)	116.4±14.8	114.9±13.5	118.0±16.3	120.9±14.6	117.2±16.3	123.6±13.7
Diastolic blood pressure (mmHg)	67.0±11.3	65.0±9.0	69.1±13.2	69.4±12.6	67.0±12.4	71.3±13.3
Heart beat (beats/minute)	75.1±9.2	74.8±6.2	75.5±11.6	78.5±10.7	75.5±5.5	80.8±13.4
Body temperature (°C)	36.5±0.4	36.5±0.3	36.5±0.4	36.6±0.4	36.4±0.3	36.7±0.4
Blood triglycerides level in fasting state (mg/dL)	81.2±26.7	79.7±25.2	82.8±28.6	101.3±23.9	104.8±20.7	98.6±27.1

^(a)^ Subgroup analysis subjects: subjects with blood triglyceride levels ≥ 200 mg/dL of C_max_ after eating the control diet.

#### Adequacy of the crossover study

A statistical significance test was performed to evaluate the effects of timing and intake order on the ΔAUC of TG. Neither a timing effect (p = 0.22) nor an order effect (p = 0.85) was observed. Therefore, the fat-loading test was considered to be appropriate based on crossover methods.

#### Evaluation of efficacy

**Fig 4:**
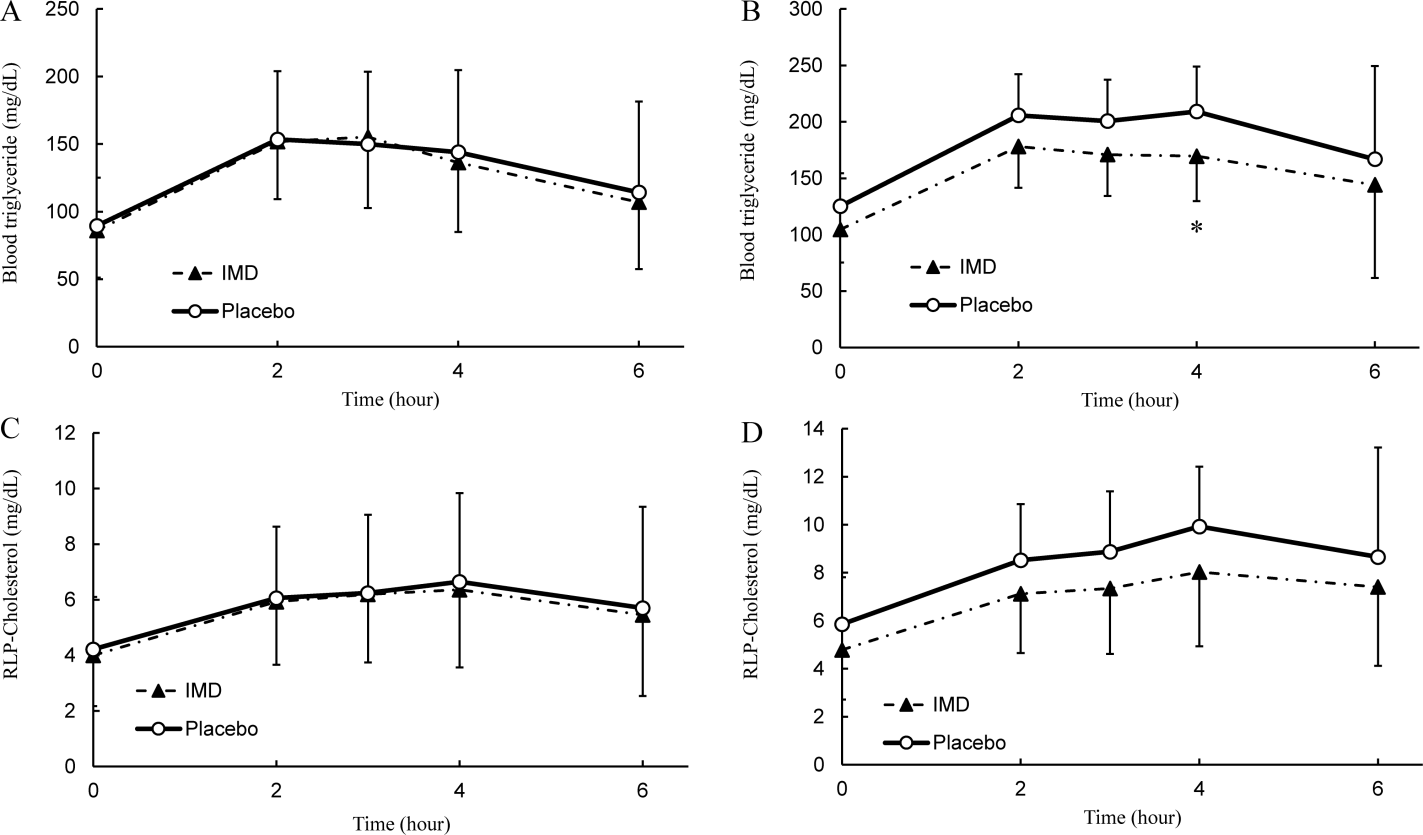
Fat-loading study in human. (A) Postprandial transition in human blood triglyceride levels (n = 40). (B) Subgroup analysis in blood triglyceride levels (n = 14). (C) Postprandial transition in human blood remnant-like particle cholesterol levels (n = 40). (D) Subgroup analysis in blood remnant-like particle cholesterol levels (n = 14). ○: Placebo, ▲: IMD. Values are mean ± standard deviation. Statistical analyses were performed using a Wilcoxon signed-rank test (*p <0.05).

The changes in blood TG levels over time are shown in Fig 4A, and the AUC, ΔAUC, C_max_ and ΔC_max_ are given in Table 2. The TG reached its maximum at 2 hours after intake of the placebo diet and 3 hours after intake of the IMD diet, and then gradually decreased until 6 hours after intake. A comparison of the TG levels between the diets showed no significant differences at 2 hours after intake. There were also no significant differences in the AUC, ΔAUC, C_max_ or ΔC_max_ between diets.

**Table 2:** C_max_ and AUC of triglyceride and blood remnant-like particle cholesterol.

		Parameter	Group	Mean±SD	*P* value
TG	All subjects	C_max_ (mg/dL)	Control	174.1±61.6	0.667
			IMD	169.4±51.9	
		AUC_0-6hr_ (mg/dL∙hr)	Control	799.7±291.4	0.978
			IMD	780.5±247.1	
		ΔC_max_ (mg/dL)	Control	84.7±42.2	0.878
			IMD	83.4±36.8	
		ΔAUC_0-6hr_ (mg/dL∙hr)	Control	263.2±155.9	0.717
			IMD	264.3±132.7	
	Subgroup analysis subjects	C_max_ (mg/dL)	Control	237.5±50.2	0.009*
			IMD	192.2±43.1	
		AUC_0-6hr_ (mg/dL∙hr)	Control	1115.1±209.9	0.084
			IMD	941.1±256.3	
		ΔC_max_ (mg/dL)	Control	112.4±50.9	0.009*
			IMD	87.7±35.0	
		ΔAUC_0-6hr_ (mg/dL∙hr)	Control	364.3±192.3	0.221
			IMD	314.1±173.3	
RLP-C	All subjects	C_max_ (mg/dL)	Control	7.2±3.5	0.540
			IMD	7.0±2.7	
		AUC_0-6hr_ (mg/dL∙hr)	Control	35.2±16.1	0.638
			IMD	34.1±13.9	
		ΔC_max_ (mg/dL)	Control	3.0±2.4	0.857
			IMD	3.0±1.7	
		ΔAUC_0-6hr_ (mg/dL∙hr)	Control	9.9±8.6	0.298
			IMD	10.1±7.1	
	Subgroup analysis subjects	C_max_ (mg/dL)	Control	10.6±3.4	0.067
			IMD	8.7±2.7	
		AUC_0-6hr_ (mg/dL∙hr)	Control	51.1±14.8	0.064
			IMD	42.3±15.3	
		ΔC_max_ (mg/dL)	Control	4.7±3.2	0.218
			IMD	3.9±2.1	
		ΔAUC_0-6hr_ (mg/dL∙hr)	Control	15.9±11.3	0.683
			IMD	13.6±9.8	

Statistical analyses were performed using a Wilcoxon signed-rank test (*p <0.05).

AUC: area under the curve, SD: standard deviation, TG: triglyceride, RLP-C: remnant-like particle cholesterol

The results of the subgroup analysis based on the C_max_ of the TG levels are shown in Fig 4B and Table 2; the analysis was performed by the same method as the overall analysis. TG levels were lower with the IMD diet than with the placebo diet at every time point. The maximum TG level with the placebo diet was reached 4 hours after intake, at which time point the TG level was significantly lower in the IMD diet than in the placebo diet (p = 0.04, r = 0.545, 95% CI = 1.50–78.50). The subjects on the IMD diet trended lower in AUC and had a significantly lower C_max_ (p = 0.009, r = 0.696, 95% CI = 13.50–70.50) and ΔC_max_ (p = 0.009, r = 0.701, 95% CI = 5.00–38.00) compared with those on the placebo diet.

The change in RLP-cholesterol levels over time is shown in Fig 4C, and the AUC, ΔAUC, C_max_ and ΔC_max_ are given in Table 2. Maximum levels of RLP-cholesterol were reached 4 hours after intake of each diet, and then gradually decreased through 6 hours after intake. Comparisons of RLP-cholesterol level at 4 hours after intakes, AUC and C_max_ between diets showed no significant differences.

Meanwhile, the results of a subgroup analysis based on C_max_ of TG are shown in Fig 4D; the analysis was performed in the same way as the overall analysis. RLP-cholesterol levels were lower in the IMD diet than in the placebo diet at every time point of measurement. The maximum level of RLP-cholesterol with the placebo diet was reached 4 hours after intake; at that time point, the RLP-cholesterol level tended to be lower in the IMD diet than in the placebo diet. The AUC and the C_max_ trended lower in the subjects on the IMD diet compared with those on the placebo diet.

#### Safety assessment

No “serious adverse events” nor adverse events related to test diets were reported at physical examination nor in the subject diary kept during the course of the study period.

### Inhibition of emulsification

**Fig 5:**
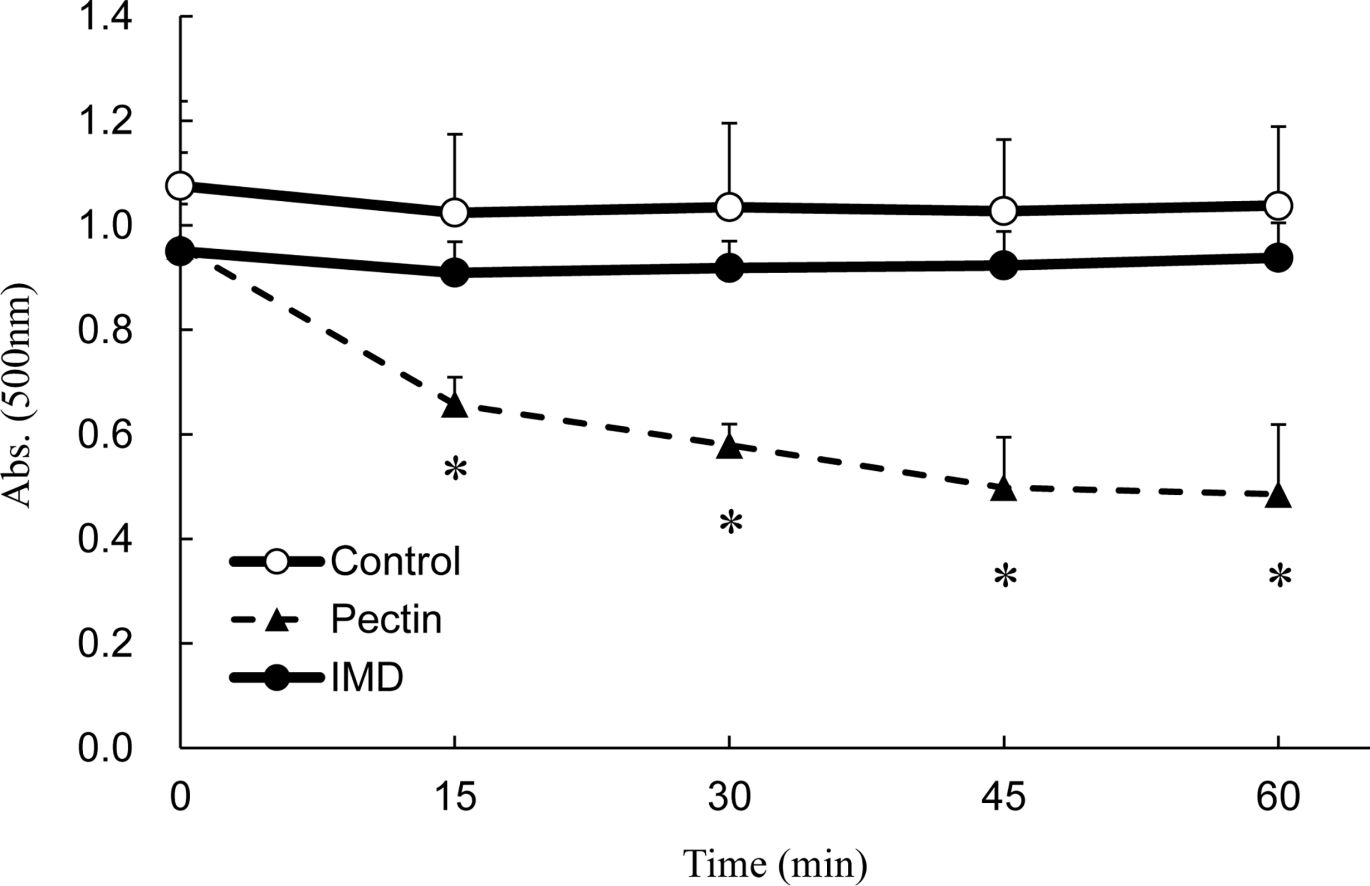
The stability of oil droplets in olive oil emulsion. Control: 0.2 M Tris-HCl buffer. Pectin: 1% pectin in 0.2 M Tris-HCl buffer. IMD: 10% IMD in 0.2 M Tris-HCl buffer. Values are mean ± standard error (n = 3). Statistical analyses were performed using Dunnett’s multiple comparison test (vs negative control: *p <0.05).

Fig 5 shows the kinetics of emulsification in terms of absorbance. When emulsification is inhibited, the absorbance will be decreased due to inhibition of particle formation, because turbidity is a measure of the number of particles in the water. Pectin, a positive control, inhibited emulsification and reduced the absorbance by approximately half by 45 minutes after addition, with significant changes compared with water, a negative control. On the other hand, IMD did not change the absorbance significantly, similar to the negative control, showing no effects on emulsification.

### Inhibition of lipase activity

**Fig 6:**
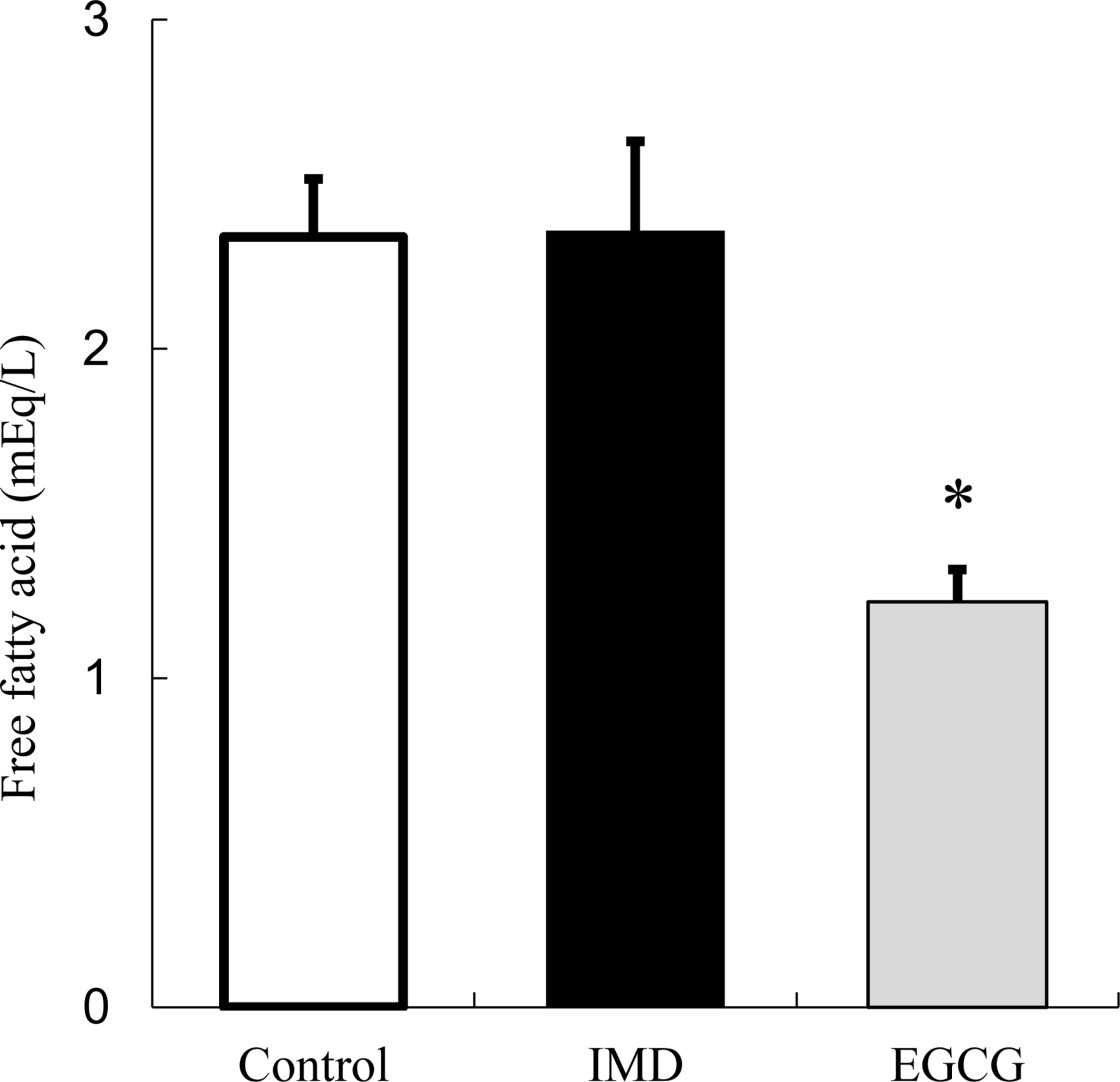
Release of fatty acids from emulsion by reaction with lipase. Control: 0.1M HEPES buffer. EGCG: 2mM EGCG in 0.1M HEPES buffer. IMD: 10% IMD in 0.1M HEPES buffer. Values are mean ± standard error (n = 3). Statistical analyses were performed using Dunnett’s multiple comparison test (vs negative control: *p <0.05).

Fig 6 shows free fatty acid concentrations. Since TG in the emulsion is cleaved by lipase, an effect on lipase activity can be observed by the change in free fatty acids. EGCG, a positive control, inhibited the production of free fatty acids by approximately half compared with a negative control not containing EGCG. On the other hand, IMD did not change the amount of free fatty acids compared with the negative control, showing no effects on lipase activity.

### Stabilization of micelles

**Fig 7:**
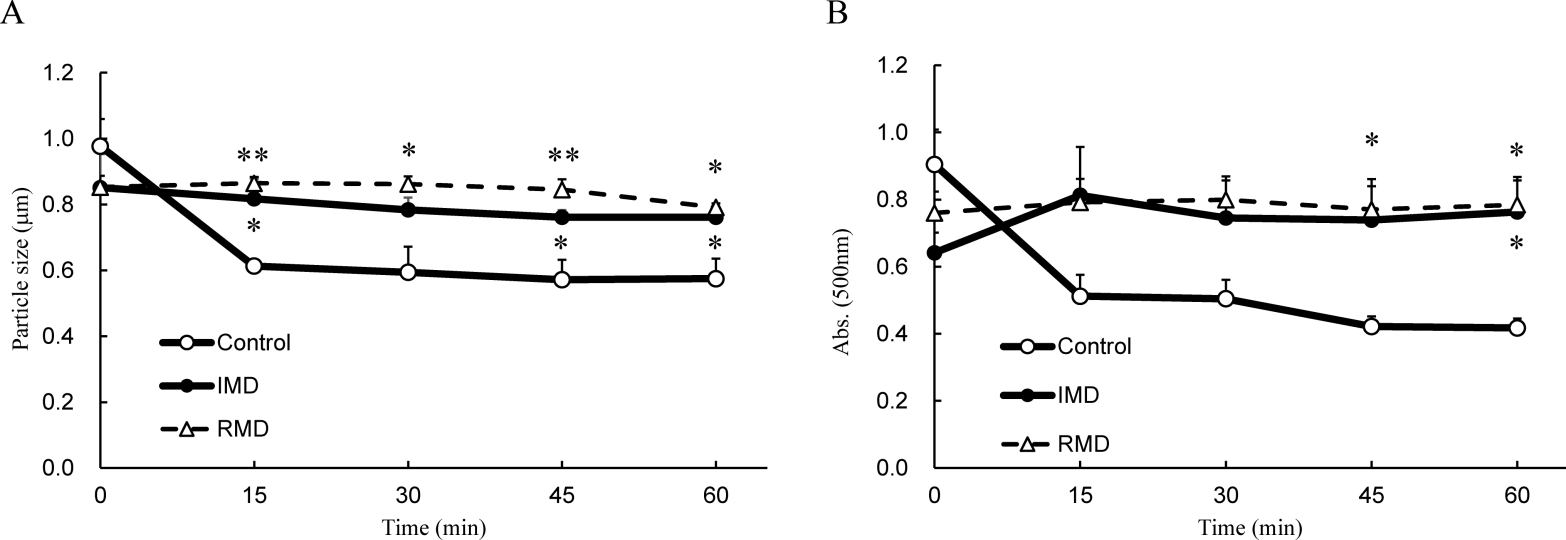
Stability of micelles. (A) Transition particle size. (B) Time transition in absorbance measured at 500 nm.;○: Control (micelle suspension prepared of water), △: RMD (micelle suspension prepared with 20% resistant maltodextrin solution instead of water), ●: IMD (micelle suspension prepared with 20% isomaltodextrin solution instead of water). Values are mean ± standard error (n = 3). Statistical analyses were performed using Dunnett’s multiple comparison test (vs negative control: *p <0.05, **p <0.01).

Fig 7 shows the change in absorbance and particle size. Degradation of particles was visualized by the reduction in absorbance and particle size in the same fashion as the inhibition of emulsification. For the negative control, rapid decreases in micelle size and absorbance were observed 15 minutes after being allowed to stand. However, for the positive control and IMD, no decrease in micelle size was observed and the absorbance was maintained, showing that IMD contributes to the micelle stability.

### Evaluation of physical properties of micelles

**Fig 8:**
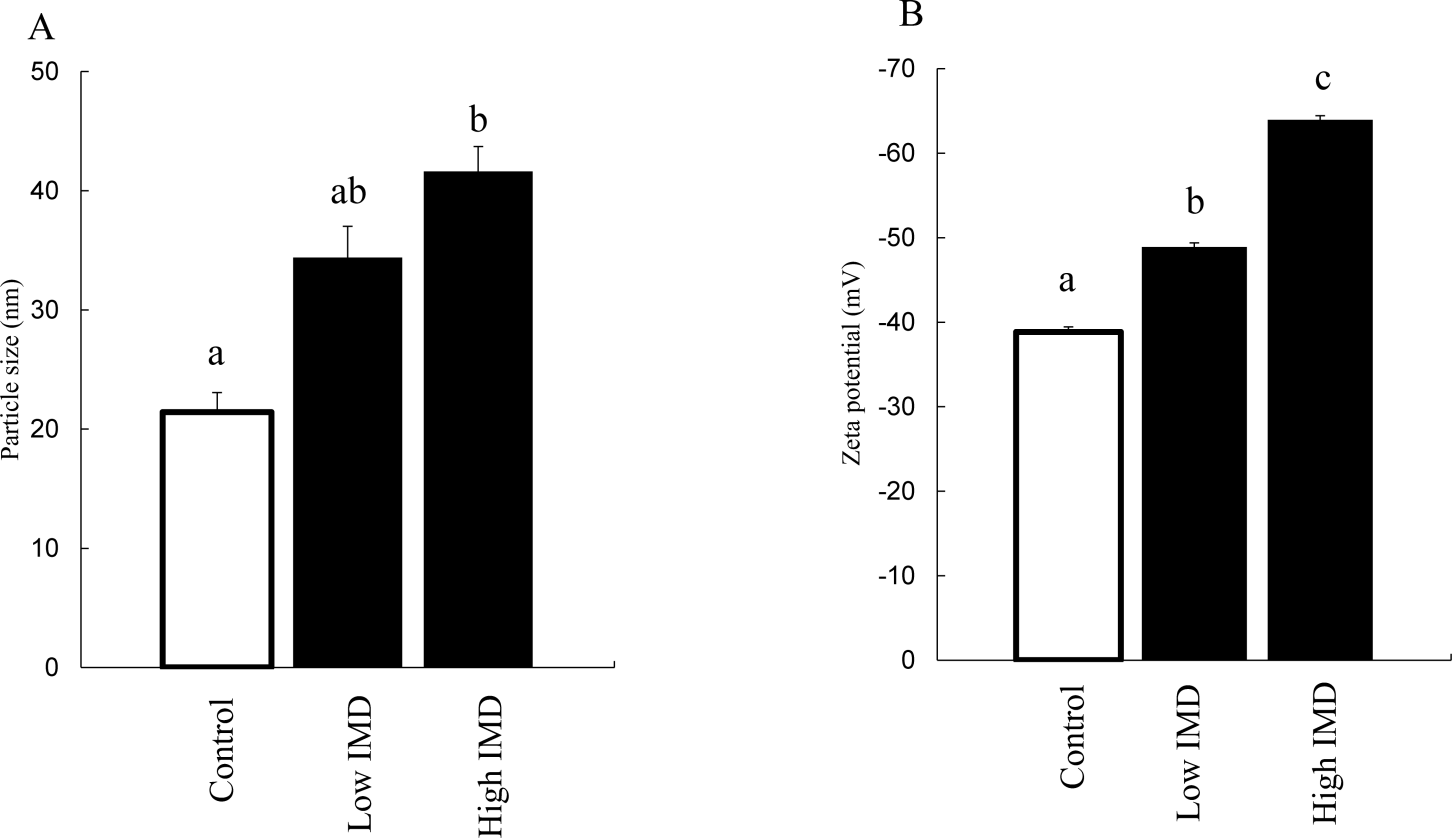
Physical properties of micelles. (A) Changes in particle size after adding IMD. (B) Changes in zeta potential after adding IMD. Control: 150 mM phosphate buffer; particle size (n = 3); zeta potential (n = 5). Low IMD: 0.1 g/mL IMD in 150 mM phosphate buffer; particle size (n = 3); zeta potential (n = 4). High IMD: 0.2 g/mL IMD in 150mM phosphate buffer; particle size (n = 3); zeta potential (n = 5). Values are mean ± standard error. Statistical analyses were performed using Tukey-Kramer multiple comparison tests. Different letters indicate significant differences: p <0.05.

Fig 8 shows the measurements of particle size and zeta potential. Since a phenomenon such as degradation does not promptly occur in a well-stabilized micelle, a change in the surface structure of the micelle particles can be observed by the changes in particle size or zeta potential. The micelle particle size was significantly enlarged in combination with a high concentration of IMD, and was slightly but not significantly enlarged with a low concentration of IMD. The zeta potential of micelles was significantly increased by IMD at high and low concentrations.

## Discussion

In this investigation, the animal studies showed that a single administration of IMD was associated with decreases in intestinal fat absorption and in elevations of blood TG levels (Fig 2). Fat absorption kinetics appeared to be directly affected by IMD, without dependence on other factors such as fermentation.

In the human study, we examined the relationship of IMD (2.5 g) intake with postprandial TG in 40 healthy men and women who were given corn soup containing 40.0 g of fat. The efficacy of IMD was evaluated by measuring TG and RLP-cholesterol before and after a high fat load. In addition, we conducted a subgroup analysis in subjects who appeared to have a high risk of abnormal lipid metabolism. The subgroup analysis was performed, based on the C_max_ of TG after receiving the placebo diet, using subjects with non-fasting TG levels ≥200 mg/dL, who are regarded as being at a high risk for abnormal lipid metabolism by the American Heart Association [21]. We considered that a significantly lower level of the ΔCmax of TG with IMD diet in the subgroup analysis is particularly meaningful despite no significant differences in TG and RLP-cholesterol between diets in the overall analysis (Table 2). These results suggest that IMD inhibits postprandial TG increases in subjects who have a normal fasting TG range but a possible high risk of abnormal lipid metabolism.

The above results suggest that the subjects included in the subgroup analysis are suitable for evaluation of the suppressive effect of IMD on postprandial TG increase. In our further studies, the establishment of inclusion criteria based on the present results may help us to make a clear evaluation. Furthermore, since there were no adverse events attributable to the test diets, IMD can be considered to be safe at the dose used in the present study.

Based on the results of animal and human studies, we investigated the mechanism of the suppression of fat absorption after IMD intake. Reported mechanisms include destabilization of emulsification in pectin [16], inhibition of lipase activity in epigallocatechin gallate [18], and stabilizing of micelles in RMD [19].

Among these mechanisms, IMD did not show destabilization of emulsification or inhibition of lipase activity (Figs 5 and 6), which are mechanisms associated with non-starch polysaccharides. IMD is a dietary fiber made from starch, and this is probably the reason why IMD was not associated with these effects.

Among dietary fibers similar to IMD that are likewise derived from starch, RMD has been reported to inhibit fat absorption by delaying the release of fatty acids from micelles through stabilizing the micelle[19].

By using the method of Kishimoto et al. [19], we examined whether IMD has a similar action mechanism to that of RMD. We found that micelles combined with IMD were stabilized similarly to RMD (Fig 7). In this method, the particle size of micelles was too large to pass through the mucin layer in the intestine[22, 23]. Therefore, it is unclear how IMD exerts its effects with the micelle size permeable enough for reaching the intestinal epithelium. Therefore, we then made use of the fact that water-soluble polysaccharides, when dissolved in water, can acquire an electrical charge due to different degrees of ionization [24], and investigated whether the surface electric charge of the micelles changed after combination with IMD. We found that the zeta potential of the micelles was significantly increased when they were combined with IMD (Fig 8). Zeta potential is a value of the potential at a given distance from the particle surface, and this value is defined as being similar to the surface electric charge. Therefore, adhesion of IMD on the particle surface may have caused an increase in the surface potential, resulting in the observed increase in zeta potential [25].

Upon mixing with IMD, the particle size of large micelles did not change (Fig 7), but the size of small micelles markedly increased (Fig 8). This action appears similar to hemagglutination, which occurs in macromolecular polysaccharides such as dextran. IMD has a structure containing many α-1,6-linkages, unlike other artificial water-soluble dietary fibers (Fig 1), and the terminal residues are similar to those of dextran [26]. Particle aggregation observed with dextran depends on the ratio of hydrodynamic radius to particle size, and it is known that particles aggregate at a large hydrodynamic radius and repulse each other at a small radius [27]. This appears to explain why IMD appeared to enlarge the small-size particles and appeared not to affect the large ones.

In the human body, ingested fat is thought to be emulsified with bile acid or phosphatide and broken down with digestive enzymes, and then delivered to and absorbed through the intestinal epithelium in the form of micro-micelles.

In this study, IMD has been shown to stabilize micron sized micelles in a manner similar to that of RMD, suggesting the inhibitory effects of IMD on the progression of micronization of micelles[19]. Micron sized particles have been reported to take longer time to pass through the mucin layer compared with nano sized particles[22]. Therefore, IMD could decrease the permeability of the mucin layer by micelles. The intestinal epithelium and mucin layer are negatively charged on the surface by acidic polysaccharide, specifically sialic acid, and surface charge can affect the permeation of anionic particles [28]. The Polyethyleneglycol has been reported to increase the surface charge by covering the particle[29]. From this, it is considered that the IMD increases the surface charge of the micelle by covering the micelle surface. As a result, IMD would be expected to increase the electrostatic repulsion between the micelles and the acidic polysaccharide and to reduce the absorption.

It is suggested that chitosan, a type of dietary fiber with basic polysaccharide property, covered the surface of micelles and inhibits the lipid absorption by changing the surface charge to the cation[30, 31]. In contrast, IMD, a type of dietary fiber with neutral polysaccharide property, could amplify the surface charge of micelles as the anion. We speculate that this mechanism of inhibition of fat absorption is also involved in the mechanism of conventional neutral dietary fiber.

Based on the above information, one of the mechanisms for the apparent inhibition of fat absorption by IMD may involve a physical property change of micelles caused by IMD localized on the micelle surface.

In this study, we focused only on TG kinetics in blood. However, this mechanism may also be applied to other lipids, and future studies of such phenomena are anticipated.

## Supporting information

S1 File Human study protocol. (PDF)

S2 File CONSORT checklist. (PDF)
