# Supplementary material for: Effects of isomaltodextrin in postprandial lipid kinetics: Rat study and human randomized crossover study
Source: PLoS One. 2018 May 1;13(5):e0196802. doi: 10.1371/journal.pone.0196802 (PMC5929559; doi:10.1371/journal.pone.0196802)
Supplement: S1 File — (PDF) [file pone.0196802.s001.pdf]

Clinical Study Protocol Synopsis

(Translation from Japanese to English)

Title of Study

The attenuating effect of substance X on postprandial blood triglyceride level in healthy human subjects.

(Site Management Organization internal control No: 16S131, Date of protocol fixation: January 19, 2017)

(UMIN study ID: UMIN000026170, Date of disclosure of the study information: February 16, 2017)

Research Institute

HAYAHIBARA CO., LTD.

Joint Research Institute

The Medical Corporation Hokubu-kai Utsukushigaoka Hospital

Site Management Organaization

Clinical Support Corporation Ltd.

Ethical Review Committee

The Ethical Committee at The Medical Corporation Hokubu-kai Utsukushigaoka Hospital

Synopsis

**Official scientific title of the study**	The attenuating effect of substance X on postprandial blood triglyceride level (TG) in healthy human subjects.
**Narrative objectives**	To evaluate the attenuating effect of substance X on postprandial TG elevation.
**Study design**	A double-blind, randomized, placebo-controlled crossover study
**Test substance**	Substance X (Isomaltodextrin powder)
**Intake method**	Intake the mixture of a high fat-loading diet and substance X.
**Subjects**	Healthy Japanese men and women aged 20 to less than 70 when consented.
**Number of subjects**	40
**Key inclusion criteria**	(1) Japanese men and women aged 20 to less than 70 when consented.<br>(2) Subjects who have a fasting TG of 30 mg/dL or more and less than 150 mg/dL.<br>(3) Subjects who are available at the study site on the visiting days.<br>(4) Subjects who agree to participate in the study and can put signature and date to the informed consent form by themselves prior to the study.
**Key exclusion criteria**	(1) Subjects with hepatic, renal, cardiac, organ disorder, diabetes, or other serious diseases.<br>(2) Subjects with surgical history of digestive system (except appendectomy).<br>(3) Subjects under medical treatment for chronic disease.<br>(4) Subjects who report that diarrhea is likely to occur when they eat high fat diets.<br>(5) Subjects who regularly take medicine, supplements and/or functional foods (including Food for Specified Health Uses [FOSHU]) that may reduce body fat or elevation of blood lipids and affect the results of the study.<br>(6) Subjects purposely taking high-fiber foods.<br>(7) Subjects who drink alcohol a lot regularly.<br>(8) Women who are pregnant or lactating or wish to become pregnant during the study.<br>(9) Subjects who have donated over 200 mL of blood or blood component within one month, or those who have donated over 400 mL of blood or blood component within three months prior to the study. Or, subjects who will donate over 1200 mL of blood or blood component during the past one year and study period, when added the planned blood sampling volume in this study.<br>(10) Subjects who judged as unsuitable for this study by the principal physician for any other reasons.
**Methods**	Forty subjects judged suitable for this study will be selected using the results of screening test.<br>This study is cross-over fashion (two-period cross-over test).<br>The subjects will be randomized into the two groups (same male-female ratio), and assigned to one group consuming the test diet first or to the other group consuming the

	control diet first. The blood will be withdrawn before the ingestion and at 2, 3, 4 and 6 hours after ingestion of the test meal. The two items which are indicators for outcomes will be measured.
**Measurement items**	Blood triglyceride level (TG).<br>Blood remnant-like particle cholesterol level (RLP-C).
**Assessment**	**Efficacy**<br>**Primary outcomes**<br>Changes in TG and the change in TG from the baseline (ΔTG) over time, and the area under the curve of TG or ΔTG; AUC calculated using TG and ΔAUC calculated using ΔTG.<br>**Key secondary outcomes**<br>Changes in RLP-C and the change in RLP-C from the baseline (ΔRLP-C) over time, and the area under the curve of RLP-C or ΔRLP-C; AUC calculated using RLP-C and ΔAUC calculated using ΔRLP-C.<br><br>**Safety**<br>Examinations of subjective and objective symptoms and adverse events by the principal physician.
**Research institute**	HAYAHIBARA CO., LTD.<br>The head of the institution : Director of R&D Center<br>Ushio Shimpei<br>Principal investigator<br>Yuki Ishida
**Joint research institute**	The Medical Corporation Hokubu-kai Utsukushigaoka Hospital<br>The head of the institution : Director of the hospital<br>Motooki Keimatsu<br>Principal investigator (Principal physician):<br>Kazuhiko Takano
**Site management organization**	Clinical Support Corporation Ltd.<br>The head of the institution : CEO<br>Toshihide Chiba<br>Study support manager :<br>Isao Takehara
**Ethical review committee**	The Ethical Review Committee at The Medical Corporation Hokubu-kai Utsukushigaoka Hospital<br>Committee chairperson:<br>Mitsuo Sato
**Planned study period**	January, 2017 – March, 2017

Agreement among Principal Investigators and SMO

In performing the study (The attenuating effect of substance X on postprandial blood triglyceride level in healthy human subjects), Principal investigator of research institute, Principal physician of joint research institute and Study support manager of site management organization agree with the contents of this study protocol.

Version Date : January 12, 2017

Research institute

Principal Investigator:

Food Materials Division, R&D Center, HAYAHIBARA CO., LTD.

Yuki Ishida

Joint research institute

Principal investigator (Principal physician):

The Medical Corporation Hokubu-kai Utsukushigaoka Hospital

Kazuhiko Takano

Site management organaization

Study support manager:

P I - Food Service Division, Clinical Support Corporation Ltd.

Isao Takehara
